# Sensored Field Oriented Control of a Robust Induction Motor Drive Using a Novel Boundary Layer Fuzzy Controller

**DOI:** 10.3390/s131217025

**Published:** 2013-12-10

**Authors:** Ali Saghafinia, Hew Wooi Ping, Mohammad Nasir Uddin

**Affiliations:** 1 UMPEDAC, University of Malaya, Kuala Lumpur 50603, Malaysia; E-Mail: wphew@um.edu.my; 2 Electrical Engineering Department, Islamic Azad University Majlesi Branch, Esfahan, 8631656451, Iran; 3 Electrical Engineering Department, Lakehead University, Thunder Bay, ON P7B 5E1, Canada; E-Mail: muddin@lakeheadu.ca

**Keywords:** variable boundary layer approach, boundary layer fuzzy controller, integral filter, speed control, induction motor keyword, current transducer sensor, position sensor

## Abstract

Physical sensors have a key role in implementation of real-time vector control for an induction motor (IM) drive. This paper presents a novel boundary layer fuzzy controller (NBLFC) based on the boundary layer approach for speed control of an indirect field-oriented control (IFOC) of an induction motor (IM) drive using physical sensors. The boundary layer approach leads to a trade-off between control performances and chattering elimination. For the NBLFC, a fuzzy system is used to adjust the boundary layer thickness to improve the tracking performance and eliminate the chattering problem under small uncertainties. Also, to eliminate the chattering under the possibility of large uncertainties, the integral filter is proposed inside the variable boundary layer. In addition, the stability of the system is analyzed through the Lyapunov stability theorem. The proposed NBLFC based IM drive is implemented in real-time using digital signal processor (DSP) board TI TMS320F28335. The experimental and simulation results show the effectiveness of the proposed NBLFC based IM drive at different operating conditions.

## 1. Introduction

Vector control techniques with sensors or sensorless are very common in induction motor control applications due to their traditional superiority in high-performance applications. With the invention of the vector control technique the AC motor became popular for variable speed drives and motion control [1]. In indirect vector control, flux and torque are decoupled under estimation of the slip speed with appropriate information about the rotor time constant. The accuracy of motor parameters, particularly, the rotor time constant plays an important role for the accuracy of the indirect vector method [2]. In order to cope with that, recently, variable-structure control (VSC), and in particular, sliding-mode control (SMC) systems [3–6], have been applied for electric motor drives.

The SMC-based drive system has many attractive features [7] such as: (1) it is robust to parameter variations and model uncertainties are insensitive to external load disturbances; (2) it offers a fast dynamic response, and stable control system; (3) it can handle some nonlinear systems that are not stable by using a linear controller; and, (4) it only requires an easy hardware/software implementation. However, due to discontinuous nature, it has some limitations in electrical drives and shows high-frequency oscillations as chattering characteristics. This chattering produces various undesirable effects such as current harmonics and torque pulsations [8,9]

In recent years, the chattering issue has become the research focus of many scholars [10–12]. Generally, introducing a thin boundary layer around the sliding surface can solve the chattering problem by interpolating a continuous function inside the boundary layer of the switching surface [13,14]. However, the slope of the continuous function is a compromise between control performance and chattering elimination [15]. Also, asymptotic stability is not guaranteed and may cause a steady-state error [16].

To improve tracking performance considering the thin boundary layer near the sliding surface, the slope of the continuous function or boundary layer thickness is adjusted by the fuzzy inference system [17,18], which is called hereafter the conventional boundary layer fuzzy controller (BLFC). However, the authors in these works did not test the performance of IM drives with large disturbances, when the controller gets saturated and the performance of the device degrades. The IM drive often faces the possibility of large uncertainties, including large external load disturbances and variations of critical motor parameters in real-time. For large disturbances, the controller needs a high gain of the reaching control part and a thicker boundary layer to eliminate the chattering effects. On the other hand, increasing the boundary layer thickness decreases the feedback system to a system without sliding mode [19]. Hence, the conventional BLFC controller is not able to completely eliminate the chattering while it improves the tracking performance of the systems with the possibility of large uncertainty.

This paper applies a modified fuzzy controller to adjust the thickness of the boundary layer near the sliding surface for improving tracking performance under small uncertainties. Also, an integral filter is proposed in the variable thin boundary layer to eliminate the chattering despite large uncertainties so that the stability of the proposed NBLFC is guaranteed. The performance of the proposed NBLFC-based IM drive is tested in both simulations and experiments and also compared with the conventional BLFC and PI controller-based IM drives.

The indirect field-oriented control (IFOC) along with the rapid progress of power electronics, DSP, sensors, and control theory can be used in high performance drive applications [20]. However, one of the challenges in real-time drive applications is the presence of noise which corrupts the useful information in measurements such as current and position/speed sensors [21–23]. It leads to the field-orientation detuning which causes degradation of the IM drive performance, so physical sensors have a key role in implementation of real-time vector control for an induction motor (IM) drive and they need to be applied properly due to their characteristics [24]. Therefore, an important requirement in an electric drive scheme is reducion the noise on any signals coming from the sensors, so this paper attempts to handle the mentioned issue by the appropriate utilization of the current and position sensors for the real-time implementation in an IM drive.

## 2. Mathematical Model of an IM for Sensored Vector Control

The mathematical models of an IM in d-q synchronously rotating reference axis are shown in Equations (1) and (2) [2]:

(1) $$\left[\begin{array}{l} v_{ds}^{e}\\ v_{qs}^{e}\\ 0\\ 0 \end{array}\right]=\left[\begin{array}{cccc} R_{s}+\sigma L_{s}p & -\sigma L_{S}\omega_{e} & \frac{L_{m}}{L_{r}}p & -\frac{L_{m}}{L_{r}}\omega_{e}\\ \sigma L_{S}\omega_{e} & R_{s}+\sigma L_{s}p & \frac{L_{m}}{L_{r}}\omega_{e} & \frac{L_{m}}{L_{r}}p\\ -L_{m}\frac{R_{r}}{L_{r}} & 0 & \frac{R_{r}}{L_{r}}+p & -\omega_{sl}\\ 0 & -L_{m}\frac{R_{r}}{L_{r}} & \omega_{sl} & \frac{R_{r}}{L_{r}}+p \end{array}\right]\;\left[\begin{array}{l} i_{ds}^{e}\\ i_{qs}^{e}\\ \phi_{dr}^{e}\\ \phi_{qr}^{e} \end{array}\right]$$

(2) $$T_{e}=\frac{3}{2}\frac{\text{P}}{2}\frac{L_{m}}{L_{r}}(i_{qs}^{e}\varphi_{dr}^{e}-i_{ds}^{e}\varphi_{qr}^{e})$$

where:

(3) $$\varphi_{dr}^{e}=({\text{L}}_{\text{m}}i_{ds}^{e}+{\text{L}}_{\text{r}}i_{dr}^{e})$$

(4) $$\varphi_{\text{qr}}^{\text{e}}=({\text{L}}_{\text{m}}i_{\text{qs}}^{\text{e}}+{\text{L}}_{\text{r}}i_{\text{qr}}^{\text{e}})$$

Rotor voltage equations in Equation (1) can be rewritten as:

(5) $$p\varphi_{dr}^{e}+\frac{R_{r}}{L_{r}}\varphi_{dr}^{e}-\frac{L_{m}}{L_{r}}R_{r}i_{ds}^{e}-\omega_{sl}\varphi_{qr}^{e}=0$$

(6) $$p\varphi_{qr}^{e}+\frac{R_{r}}{L_{r}}\varphi_{qr}^{e}-\frac{L_{m}}{L_{r}}R_{r}i_{qs}^{e}+\omega_{sl}\varphi_{dr}^{e}=0$$

In IFOC, the rotor flux is oriented entirely in the d-axis by setting $\varphi_{qr}^{e}=0$, so:

(7) $$\varphi_{r}^{e}=\varphi_{dr}^{e}$$

By substituting Equation (5) in Equations (6) and (7), the slip frequency is obtained as:

(8) $$\omega_{sl}=\frac{L_{m}}{\varphi_{r}^{e}}\left(\frac{R_{r}}{L_{r}}\right)i_{qs}^{e}$$

Considering $\varphi_{qr}^{e}=0$ Equation (5) can be shown in the steady state as:

(9) $$\varphi_{dr}^{e}=L_{m}i_{ds}^{*e}$$

Considering Equations (2) and (7), and $\varphi_{qr}^{e}=0$ the torque is obtained as,

(10) $$T_{e}=\frac{3}{2}\frac{\text{P}}{2}\frac{{L_{m}}^{2}}{L_{r}}i_{qs}^{*e}i_{ds}^{*e}=K_{t}i_{qs}^{*e}$$

where:

(11) $$K_{t}=\frac{3}{2}\frac{\text{p}}{2}\frac{{L_{m}}^{2}}{L_{r}}i_{ds}^{e*}$$

**Figure 1.**
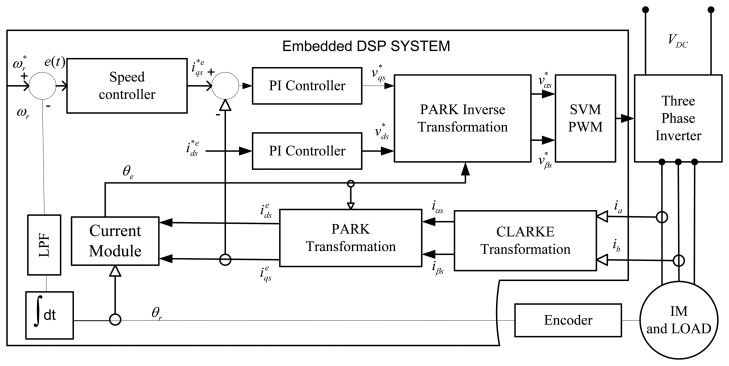
Block diagram of a closed loop sensord IFOC based IM drive.

**Figure 2.**
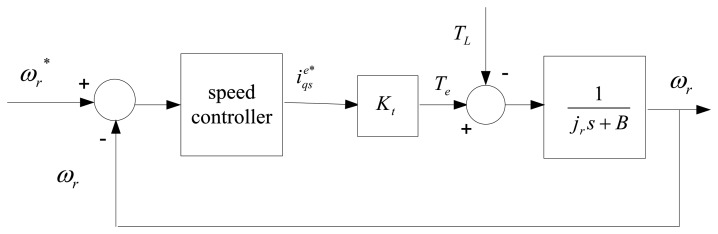
Block diagram of simplified IFOC of IM.

Considering the implementation of sensored field-oriented control as shown Figure 1, the IM drive can be simplified as Figure 2 [25]. The mechanical equation of an induction motor can be presented as follows:

(12) $$J_{r}\overset{•}{\omega_{r}(t)}+B\omega_{r}(t)=T_{e}-T_{L}$$

where *J_r_*, *B* and *T_L_* are represented as rotor inertia, friction factor and the external load disturbance, respectively. Substituting Equations (10) and (11) in Equation (12) yields:

(13) $$\overset{•}{\omega_{r}(t)}=-\frac{B}{J_{r}}\omega_{r}(t)+\frac{k_{t}}{J_{r}}i_{qs}^{*e}-\frac{T_{L}}{J_{r}}=B_{p}\omega_{r}+A_{p}i_{qs}^{*e}+D_{p}T_{L}$$

where, *A_p_* = *k_t_* / *J_r_* > 0, *B_p_* = −*B* / * J_r_* < 0 and *D_p_* = −1 / *J_r_* > 0.

To achieve the nominal model of an IM drive, the nominal value of the parameters must be considered without any disturbances [26]. Thus, the nominal model of the IM drive given by Equation (13) can be written as:

(14) $$\overset{•}{\omega_{r}(t)}=\bar{B}\omega_{r}+\bar{A}i_{qs}^{*e}$$

where, *A̅* = *k̅_t_* / *J̅_r_* and *B̅* = −*B̅* / * J̅_r_* are the nominal values of A*_P_* and B*_p_*, respectively. To handle the uncertainties, they must be considered and added to the nominal model for real-time induction motor (IM) drive. So, the dynamic Equation (14) considering structured and unstructured uncertainties and the unmodeled dynamics for the actual IM drive is obtained as:

(15) $$\overset{•}{\omega_{r}(t)}=\bar{(B}+\Delta B)\omega_{r}(t)+(\bar{A}+\Delta A)i_{qs}^{*e}+D_{p}T_{L}+\delta =\bar{B}\omega_{r}(t)+\bar{A}i_{qs}^{*e}+L(t)$$

where, $L(t)=\Delta B\omega_{r}(t)+\Delta Ai_{qs}^{*e}+DT_{L}+\delta$

In the above equation, the uncertainties are shown by ΔA and ΔB. Also unstructured uncertainty due to detuning field-orientation in the transient state and the unmodeled dynamics in practical applications are shown as *δ*. In the above equation, *L*(*t*) is called lumped uncertainty and it is assumed that the bound of $\overset{•}{L\left(t\right)}$ is unknown but is limited as $\left|\overset{•}{L\left(t\right)}\right|<m$ where, *m* is a positive constant.

## 3. The Conventional SMC-Based Controller

Considering the speed tracking error, *e*(t) = *ω_r_*(t) − ω*_r_(t), time-varying surface of sliding mode in the state of space *R^2^* is introduced as below:

(16) $$S(t)=h\left(Ce(t)+\overset{•}{e(t)}\right)$$

where *C* and *h* in above scalar equation is a strictly positive constant. Substituting Equation (10) and Equation (11) in Equation (12) without consideration of lumped uncertainty ( $\overset{•}{L\left(t\right)}=0$), the desired performance under nominal system model (equivalent control) can be achieved [14] as shown in Equation (18):

(17) $$\overset{•}{S(t)}=h\left(C\overset{•}{e(t)}+\bar{B}\overset{•}{\omega_{r}(t)}+\bar{A}u(t)+\overset{•}{L(t)}-\overset{•}{\omega_{r}^{*}}(t)\right)=0$$

where: $u(t)=\overset{•}{i_{qs}^{e}}(t)$.

(18) $$u_{eq}(t)=-{\left(\bar{A}\right)}^{-1}[(C+\bar{B})\overset{•}{e(t)}+\bar{B}\overset{•}{\omega_{r}^{*}}(t)-\overset{••}{\omega_{r}^{*}}(t)]$$

In order to achieve suitable performance despite uncertainties on the dynamic of the system (lumped uncertainty), a discontinuous term must be added to equivalent control part across the sliding surface *S*(*t*). The term discontinuous is called hitting control part or reaching control part of control effort [14]. It is given as:

(19) $$u_{r}(t)=-{\left(\bar{A}h\right)}^{-1}k(t)\mathrm{sgn}\left[S(t)\right]$$

where, *k*(*t*)> 0 and “sgn” is the sign function as below.

(20) $$\mathrm{sgn}\left[S(t)\right]=\{\begin{array}{ccc} 1 & if & S(t)>0\\ -1 & if & S(t)<0 \end{array}$$

Therefore, favorable control performance considering uncertainties and unmodeled dynamics can be achieved by the control law as below:

(21) $$u(t)=u_{eq}(t)+u_{r}(t)$$

(22) $$i_{qs}=\frac{1}{\tau }\int_{0}^{t}u(t)dt$$

where, *τ* is the integral constant. By defining the Lyapunov function as:

(23) $$V(t)=\frac{1}{2}S^{2}(t)$$

Stability condition can be obtained from the Lyapunov stability theorem as [27]:

(24) $$\overset{•}{V}(t)=S(t).\overset{•}{S(t)}\leq -\eta \left|S(t)\right|$$

where, *η* is a strictly positive constant. Substituting Equations (16) and (17) in Equation (24) and considering $\left|\overset{•}{L(t)}\right|<m$, stability of the system is guaranteed by the following equation:

(25) k(t)≥hm+η

## 4. The Proposed NBLFC Controller

Essentially, eliminating chattering phenomenon is done by smoothing out the control discontinuity in a thin boundary layer near to the sliding surface [27]. To eliminate the chattering and improve the tracking performance under small uncertainties, the boundary layer thickness *ψ* is changed using the fuzzy inference system [17,18]. In fact, the sign function in Equation (18) is replaced by a “saturation ” function as follows:

(26) $$\text{sat}\left[S(t)/\psi (t)\right]=\{\begin{array}{ccc} \frac{S(t)}{\psi (t)} & if & \left|S\right|\leq \left|\psi (t)\right|\\ \mathrm{sgn}\left[S(t)\right] & if & \text{othewise} \end{array}$$

where, *ψ*(t) is defined as the variable boundary layer thickness. In this state, to ensure asymptotic stability, the stability condition must be modified due to the boundary layer thickness changes [14]. Then, the stability condition Equation (23) and reaching control part Equation (18) have been changed as shown in Equations (27) and (28), respectively:

(27) $$\begin{array}{ccc} \overset{•}{V}\leq -(\eta -\overset{•}{\psi })\left|S(t)\right| & \text{for} & \left|S(t)\right|\geq \text{Ψ}(t) \end{array}$$

(28) $$u_{r}(t)=-{\left(\bar{A}h\right)}^{-1}\bar{k}(t)\text{sat}\left[S(t)/\psi (t)\right]$$

where, *k̅*(*t*) = *k*(*t*) − *ψ̇*(*t*).

Overall, heuristic techniques are usually complex which tends to mask the simplicity of fuzzy control and contribute to time delays when attempted for real-time control [28]. Therefore, this work attempts to reduce the high computational burden resulting in the time delay in real-time by taking some points into account to design the fuzzy controller discussed as follows.

The design of the fuzzy controller essentially consists of a knowledge-based design that includes formulation of membership function (MF) shape and its distribution of the fuzzy variables, the rule matrix design, and a number of linguistic rules. It can be shown that MFs play an important role in the performance of fuzzy control systems. *A priori* determination of membership function shape and its optimum distribution is the best for fast, simple and effective design of fuzzy controllers [28]. The triangular type MF is the best for fuzzy controlled drive systems [28,29], because the triangular membership function gives a reasonably good performance in terms of theoretical calculations as compared to other shapes and it also has linear interpolation between fuzzy set elements [30,31], hence, the triangular membership function is applied in this work.

In addition, the most common approaches to FIS are the Sugeno and Mamdani approaches. In the Sugeno approach it would be difficult to give a linguistic interpretation of the information that is described in the rule base, while, the Mamdani approach is typically used in modeling human expert knowledge [32]. Also, a common and useful defuzzification technique is the center of area (COA) method rather than the other methods [31,33], so the Mamdani type fuzzy inferencing method and COA deffuzification method are used in this paper.

Based on the aforementioned discussion, this paper modifies an existing fuzzy controller in the literature [17] to adjust the thickness of boundary layer for improving tracking performance under small uncertainties. The existing fuzzy controller is modified so that the switching variable alteration (| Δ*S*(t)|) is added for the switching variable |*S*(t)| as input of the fuzzy inference system and the number of fuzzy membership functions (MFs) is also increased for better control of the thin boundary layer thickness. The fuzzy rules are designed to reduce the tracking errors while the reduction of the control error ends increasing the thickness of the boundary layer. Then, the boundary layer thickness as a nonlinear function of variable switching |*S*(t)| and | Δ*S*(t)| is obtained as:

(29) ψ(t)=BLFC(|S(t)|,|ΔS(t)|)

Substituting Equation (29) in Equation (28) and considering Equation (21), the control law is obtained as:

(30) $$u(t)=u_{eq}(t)-{\left(\bar{A}h\right)}^{-1}\bar{k}(t)\text{sat}\left[\text{BLFC}\left(\left|S(t)\right|,\left|\Delta S(t)\right|\right),S(t)\right]$$

**Figure 3.**
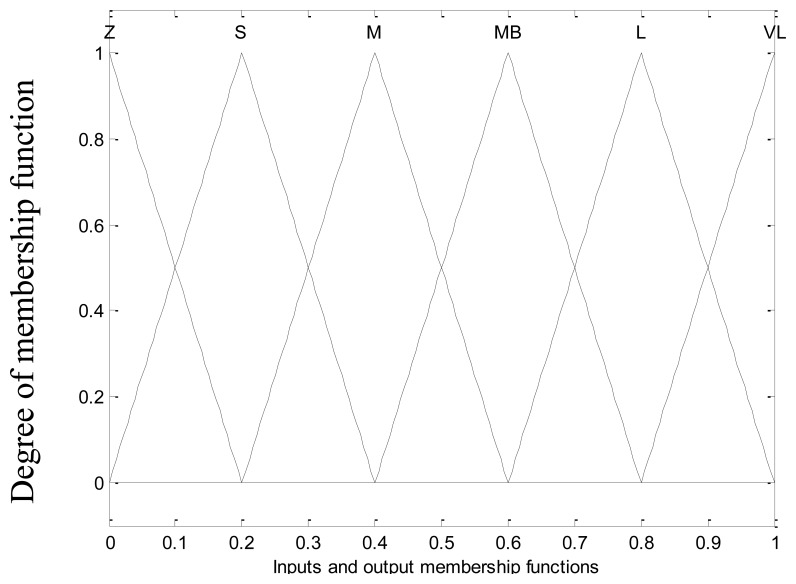
Inputs and output membership function.

The Mamdani type fuzzy inference method with 36 rules shown in Table 1 is used. The center of the area (COA) method is used as the defuzzification method. Triangular type membership functions with fuzzy sets zero (Z), small (S), medium (M), medium big (MB), large (L), and very large (VL) have been defined on the interval [0, 1] for inputs and output as shown in Figure 3. In this stage, the stability condition Equation (27) is satisfied by choosing the control law (30) and control gain as Equation (25) and consequently the stability of the system is guaranteed for |*S*(*t*)| > *ψ*(*t*).

An integral filter is designed inside the variable thin boundary layer to eliminate the chattering despite large uncertainties. To design the integral filter, the related discussions are presented as follows.

For |*S*(*t*)| ≤ *Ψ*(*t*) with substituting Equation (30) in Equation (17), yields:

(31) $$\overset{•}{S(t)}+\bar{k}(t)\frac{S(t)}{\psi (t)}=h\overset{•}{L(t)}$$

Since *k̄*(*t*) and *L̇*(*t*) are continuous in *ω_r_*, Equation (31) can be written as below:

(32) $$\overset{•}{S(t)}+{\bar{k}}_{d}(t)\frac{S(t)}{\psi (t)}=h\overset{•}{L_{d}(t)}+o(\zeta )$$

where ${\bar{k}}_{d}(t)=k_{d}(t)-\overset{•}{\psi (t)}$ and $\overset{•}{L_{d}(t)}$ is declared in desired state (ω*_r_) and O(ξ) shows the error of replacing parameter *ω_r_*(t) for ω*_r_ (t). Variable switching *S*(t) in Equation (32) as output, whose dynamics only depend on the desired state ω*_r_ (t), is passed through a first-order low pass filter whereas the lumped uncertainty is considered as input. Thus, the chattering can be omitted.

The cut-off frequency of the filter Equation (32) is obtained as:

(33) $$\upsilon =\frac{{\bar{k}}_{d}(t)}{\psi (t)}$$

In the above equation, *υ* must be less than or equal *C* [34]. The value of the tracking precision *ε* equals *ψ*(*t*)/ *υ*. Considering Equation (33) and ${\bar{k}}_{d}\left(t\right)=k_{d}\left(t\right)-\overset{•}{\psi \left(t\right)}$, the equation of *ψ*(*t*) is obtained as:

(34) $$\overset{•}{\psi (t)}+\upsilon \psi (t)=k_{d}(t)$$

The variable switching *S*(*t*) has a steady-state value that depends on the inputs of Equation (31). Therefore, to achieve a precise tracking performance despite the possibility of large uncertainties in the system, increasing the control gain might increase *S*(*t*). In this state by using the thin boundary layer, it grows until it hits the boundary layer and when it is out of the boundary layer, it is imposed inward because of boundary layer attractiveness. Hence, this effectiveness produces chattering again. In fact, the discontinuity in the sat function Equation (30) at *S*(*t*) = *ψ* causes the chattering [35]. To overcome this problem, the discontinuity value is controlled and multiplied by a coefficient *α*(*t*) so that this coefficient *α*(*t*) fixes the bandwidth filter inside the boundary layer. Thus, the amount of discontinuity limits the value of the achievable filter bandwidth. To tackle the problem at this stage, an integral action with coefficient *β*(*t*) is added to force the trajectory on both sides of the boundary layer to face inward in order to prevent the chattering, so the reaching control Equation (30) changes to:

(35) $$u_{r}(t)=-\bar{k}(t){\left(\bar{A}h\right)}^{-1}\text{fsat}\left(\left(\psi (t),S(t),\alpha (t),\beta (t\right)\right)$$

where:

(36) $$\text{fsat}\left(\left(\psi (t),S(t),\alpha (t),\beta (t\right)\right)=\{\begin{array}{c} \begin{array}{ccc} \alpha (t)\frac{S(t)}{\psi (t)}+\frac{\beta (t)}{\psi (t)}\int_{0}^{t}S(t)dt & \text{for} & \left|S(t)\right|\leq \left|\psi (t)\right| \end{array}\\ \begin{array}{ccc} \mathrm{sgn}\left[S(t)\right] & \text{for} & \left|S(t)\right|>\left|\psi (t)\right| \end{array} \end{array}$$

**Figure 4.**
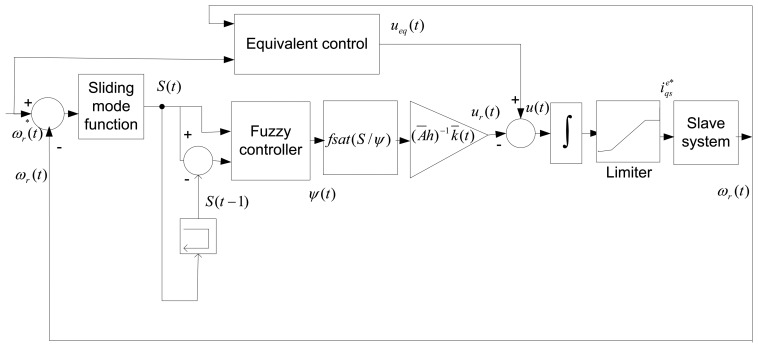
The control block diagram of the proposed NBLFC-based IM drive.

Then, the block diagram of the proposed NBLFC-based IM drive is obtained as shown in Figure 4. In this stage, stability condition Equation (27) is satisfied by choosing the reaching control Equation (35) and the control gain Equation (25) and consequently the stability of the system is guaranteed for |*S*(*t*)*| >ψ(t)*. For |*S*(*t*)*|* ≤ *ψ(t)* the system trajectory is declared in terms of the variable switching *S*(*t*) as:

(37) $$\overset{•}{S(t)}+{\bar{k}}_{d}(t)(\alpha (t)\frac{S(t)}{\psi (t)}+\frac{\beta (t)}{\psi (t)}\int_{0}^{t}S(t)dt)=h\overset{•}{L_{d}(t)}+o(\zeta )$$

In the above equation, *α*(*t*) and *β*(*t*) are chosen so that *S*(*t*) → 0 for *t* → ∞. Then, they can be chosen as follows:

(38) $$\alpha (t)=\frac{2\upsilon \psi (t)}{{\bar{k}}_{d}(t)}$$

(39) $$\beta (t)=\frac{\upsilon^{2}\psi (t)}{{\bar{k}}_{d}(t)}$$

In this state, the equation of *ψ*(*t*) is obtained as:

(40) $$\overset{•}{\psi (t)}+2\upsilon \psi (t)=k_{d}(t)$$

Substituting Equations (38) and (39) in Equation (37) and considering Equation (33), Equation (37) represents a filter as:

(41) $$\overset{•}{S(t)}+2\upsilon \left(S(t)+\upsilon^{2}\int_{0}^{t}S(t)dt\right)=h\overset{•}{L_{d}(t)}+o(\zeta )$$

The polynomial roots of the filter above are obtained as:

(42) $$\frac{1}{p}\left(p^{2}+2\upsilon p+\upsilon^{2}\right)=0$$

**Figure 5.**
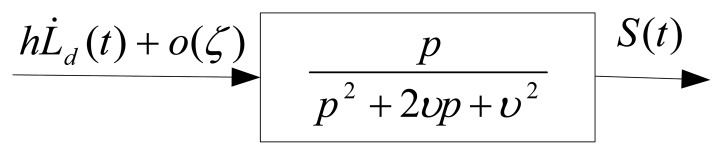
The integral filter inside boundary layer.

In the above filter (41), the lumped uncertainty as input and *S*(*t*) as output are considered as shown in Figure 5. Thus, with choosing *α*(*t*), *β*(*t*) as Equation (38), Equation (39) respectively, and the integral filter as Equation (41), the stability system is guaranteed and the chattering phenomenon is eliminated completely with the possibility of large uncertainty.

## 5. Simulation and Experimental Results

### 5.1. Simulation Results and Discussion

The performance of the proposed NBLFC-based IM drive has been investigated extensively in simulation. In order to show the superiority the performance of the proposed NBLFC is also compared with the tuned PI and the conventional BLFC controllers under different operating condition such as a sudden change of load, change of command speed, and parameter changes.

**Figure 6.**
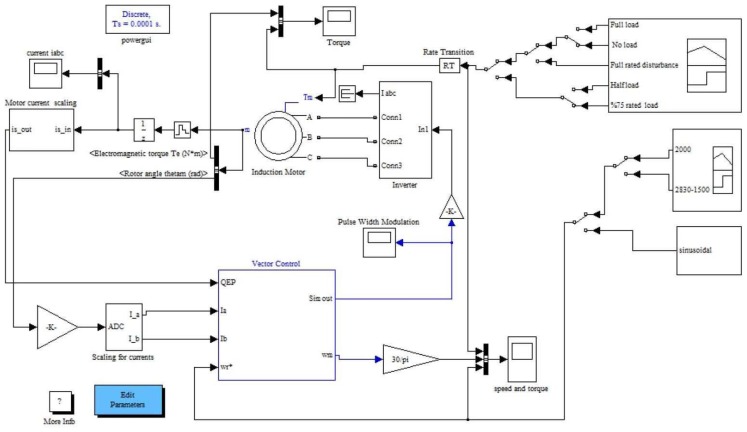
The main Simulink model for the IFOC of an IM drive.

The discrete time Simulink model with sampling time Ts = 1 × 10^−4^ s along with the digital motor control (DMC) and IQMath libraries from TI and Mathworks are used to simulate the IFOC induction motor drive. These libraries were used to optimize the Simulink blocks. The SVM-VSI type inverter is modelled based on the fast switching IGBTs model from the Simulink toolbox along with the aforementioned libraries in MATLAB. Based on the block diagram of closed-loop vector control of the IM drive shown in Figure 1, a complete Simulink block diagram is designed, which is shown in Figure 6. TI and Mathworks provide a library of highly optimized and high precision math functions in the form of the IQMath library. IQMath is a way of representing a numeric value containing a sign, the integer and the fraction portion in a fixed bit location field. The IQ Math library is available in both fixed-and floating-point versions, enabling easy migration from float to fixed devices. These tools enable developers to quickly determine the processing resources required to implement basic motor control. The PI controller is tuned by the Ziegler–Nichols method based on the stability boundary [36] to get the minimum overshoot/undershoot, minimum settling time and no steady-state error. The PI parameters are found as, Kp = 0.3, Ki = 0.0001, and Kc = 0.0001. The parameters of the IM are listed in Table 2.

For simulation tests, the following cases including parameter variations and external load disturbance are considered. If not mentioned, all other parameters are considered nominal in all the cases:

***Case-1***: Step changes in command speed with no load
***Case-2***: Step increase in load from ‘0’ to 75% of rated load at t = 7 s.
***Case-3***: Inertia and friction coefficient are increased two times of nominal value at t = 7 s while half rated load is applied from the beginning.

Within the restriction of the control effort, in order to achieve the system stability, and the best transient performance, the control parameters are chosen as, *C* = 1,500, *h* = *Ā^-1^*, *τ* = 1, *K* = 220, and *υ* =0.003.

**Figure 7.**
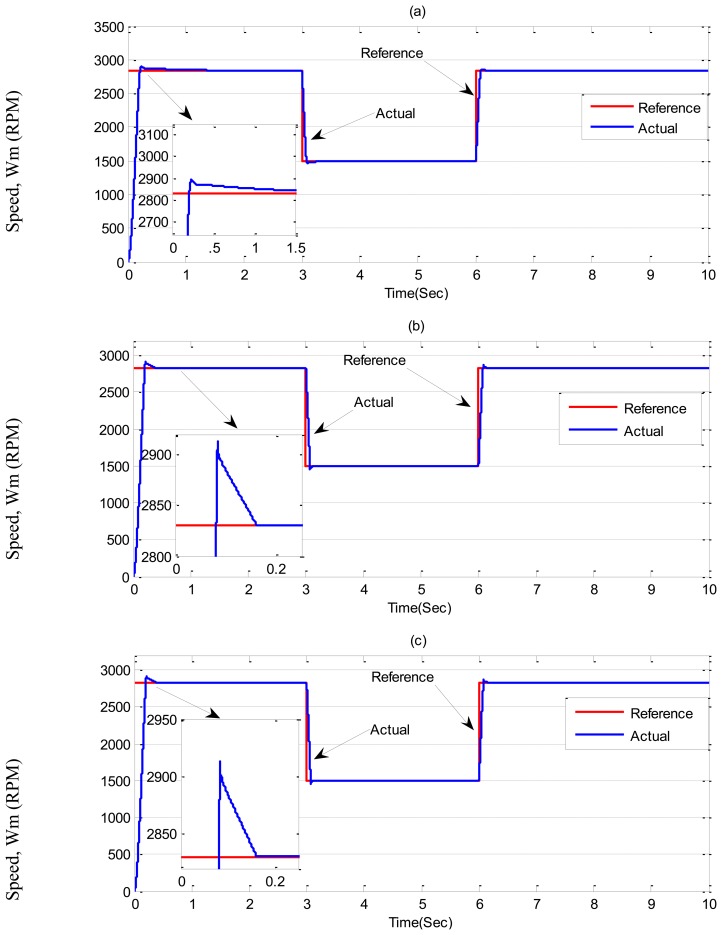
Simulated speed responses-based IM drive at no load in *Case-1*; (**a**) the tuned PI controller, (**b**) the conventional BLFC controller, and (**c**) the proposed NBLFC controller.

**Figure 8.**
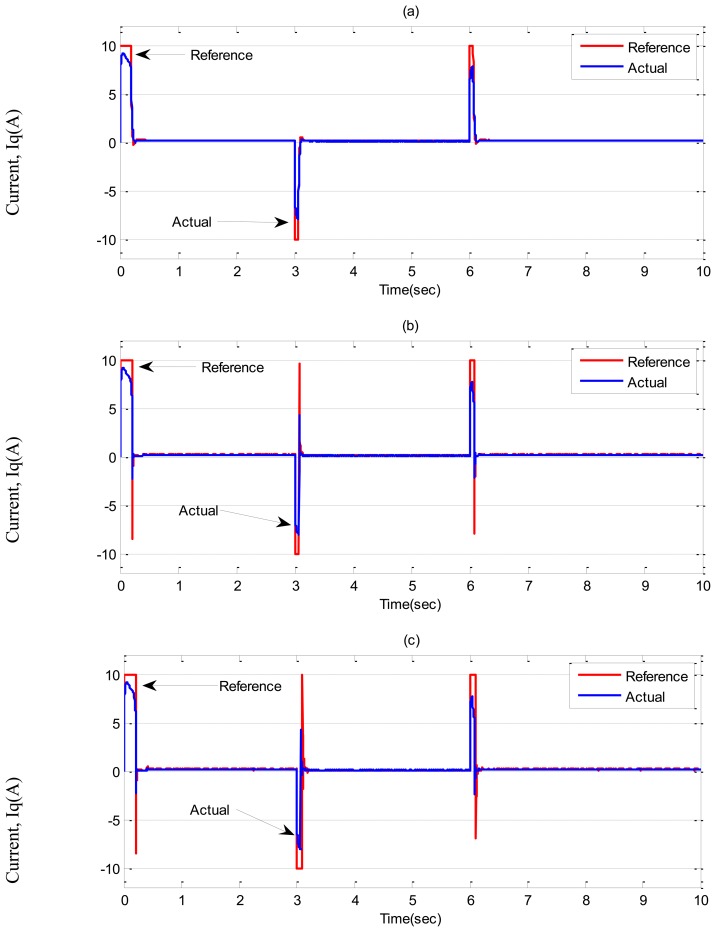
Simulated q-axis current responses-based IM drive at no load in *Case-1*; (**a**) the tuned PI controller, (**b**) the conventional BLFC controller, and (**c**) the proposed NBLFC controller.

In ***Case-1***, simulation results are illustrated in Figures 7 and 8 for the tuned PI, conventional BLFC and the proposed NBLFC controllers, respectively. From Figure 7, it can be seen that dynamic performance of the proposed NBLFC controller is almost similar to the conventional BLFC and better than those of the tuned PI controller. It can also be seen from Figures 7 and 8 that the conventional BLFC and the proposed NBLFC do not have any chattering in speed while maintaining almost the same tracking response.

**Figure 9.**
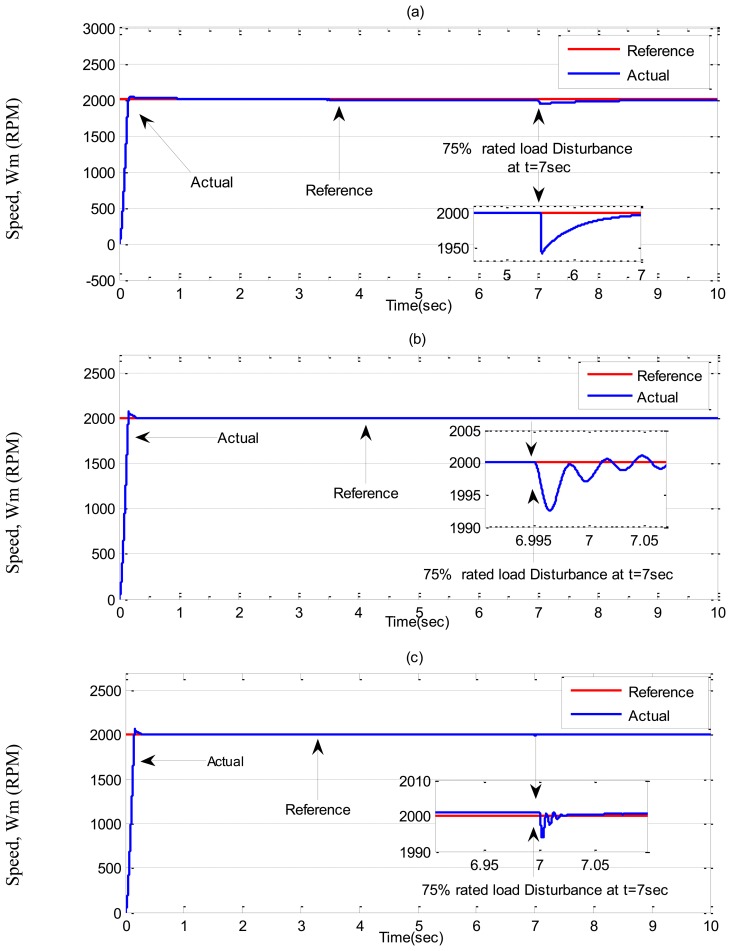
Simulated speed responses-based IM drive in *Case-2*; (**a**) the tuned PI controller, (**b**) the conventional BLFC controller, and (**c**) the proposed NBLFC controller.

**Figure 10.**
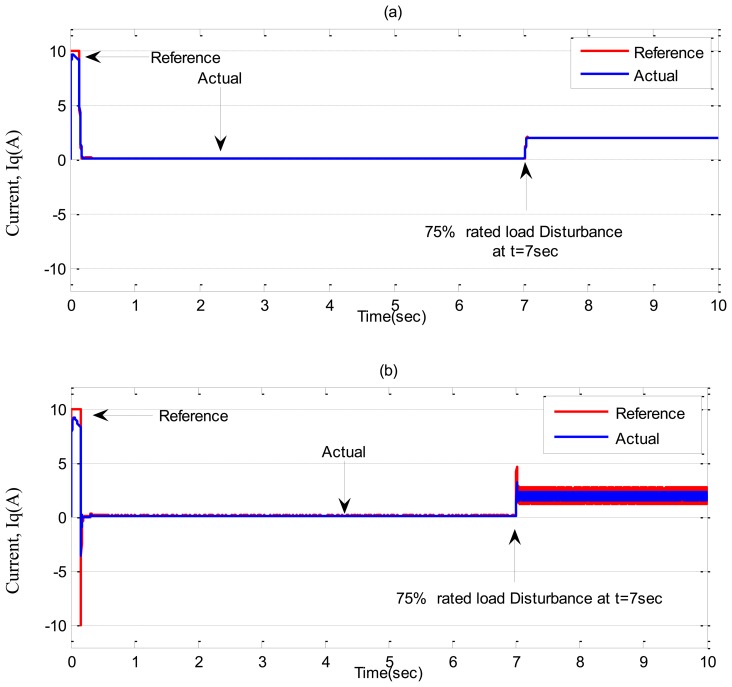

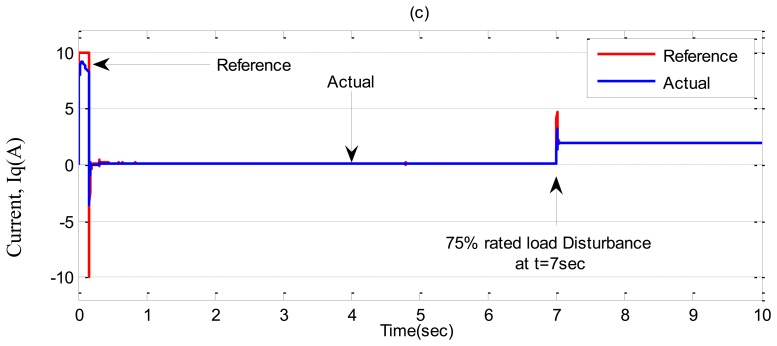
Simulated q-axis current responses-based IM drive in *Case-2*; (**a**) the tuned PI controller, (**b**) the conventional BLFC controller, and (**c**) the proposed NBLFC controller.

Simulation results for the tuned PI, conventional BLFC and the proposed NBLFC controllers, respectively, in ***Case-2*** are shown in Figures 9 and 10. It can be seen the PI controller suffers from a significant dip in speed (≈60 RPM) when the step increase in load is applied at t = 7 s. On the other hand, the conventional BLFC and the proposed NBLFC controllers are found to be almost insensitive when a step increase in load is applied. It is also found that the conventional BLFC suffers from chattering only when an external load disturbance is applied, whereas, it is found from Figures 9 and 10 that the proposed NBLFC is not only insensitive to load variations but also free from chattering in steady-state despite large uncertainty in the system. Moreover, the settling time for the proposed NBLFC based IM drive is faster than that of the tuned PI and almost similar to the conventional BLFC controller. Thus, the proposed NBLFC based IM drive ensures smooth operation of the motor and less harmonic losses in the motor.

**Figure 11.**
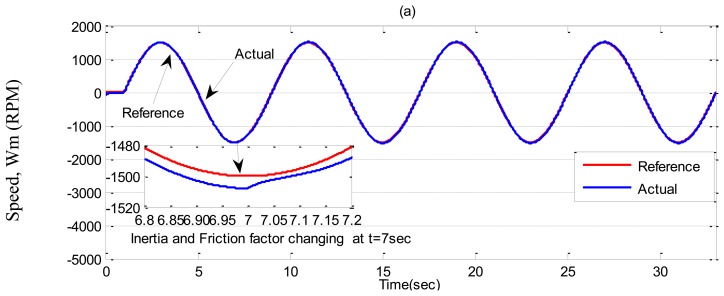

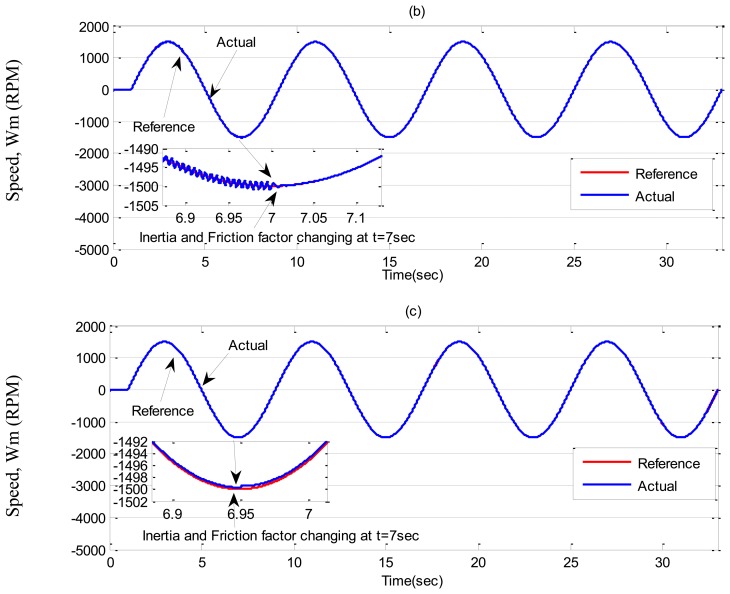
Simulated speed responses based IM drive in *Case-3*; (**a**) the tuned PI controller, (**b**) the conventional BLFC controller, and (**c**) the proposed NBLFC controller.

**Figure 12.**
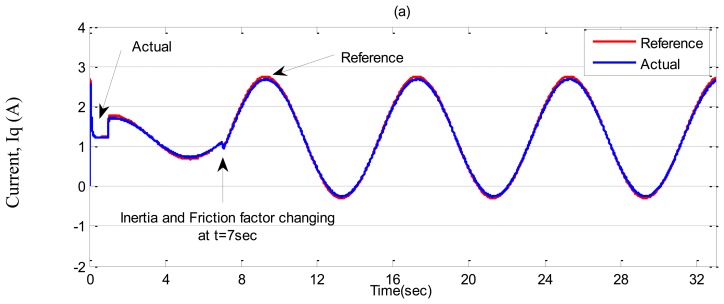

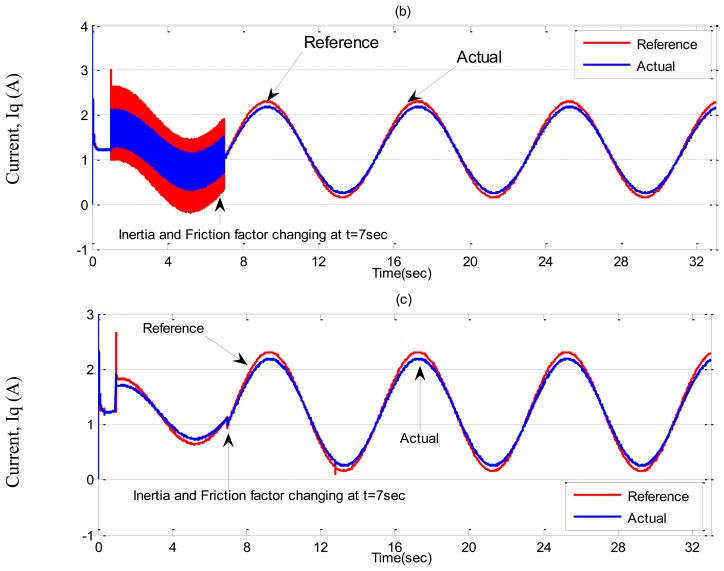
Simulated q-axis current responses-based IM drive in *Case-3*; (**a**) the tuned PI controller, (**b**) the conventional BLFC controller, and (**c**) the proposed NBLFC controller.

**Figure 13.**
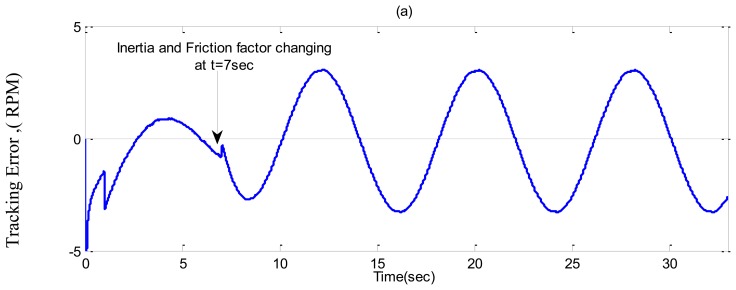

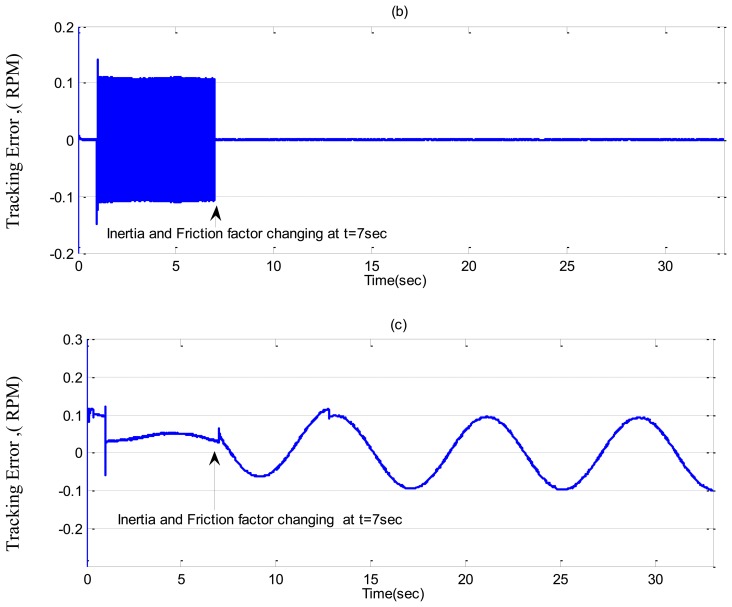
Simulated tracking error responses-based IM drive in *Case-3*; (**a**) the tuned PI controller, (**b**) the conventional BLFC controller, and (**c**) the proposed NBLFC controller.

In ***Case-3***, a sinusoidal command speed is selected to properly show the tracking error. The other parameter variations are also tested in this case. Simulation results are shown in Figures 11, 12 and 13 for the tuned PI, the conventional BLFC, and the proposed NBLFC, respectively. It is found from these figures that the tracking capability of the conventional BLFC and proposed NBLFC is much better than the tuned PI controller. It is also found from these figures that the proposed NBLFC has acceptable tracking performance without any chattering, despite the large uncertainty in the system, while the conventional BLFC suffers from chattering for both speed and current. Due to the damping effect the conventional BLFC showed better response without chattering when the inertia and friction coefficient are increased. Thus, the highest accuracy in speed tracking of the proposed NBLFC with almost no chattering in current is verified in our simulation.

### 5.2. Experimental Implementation

**Figure 14.**
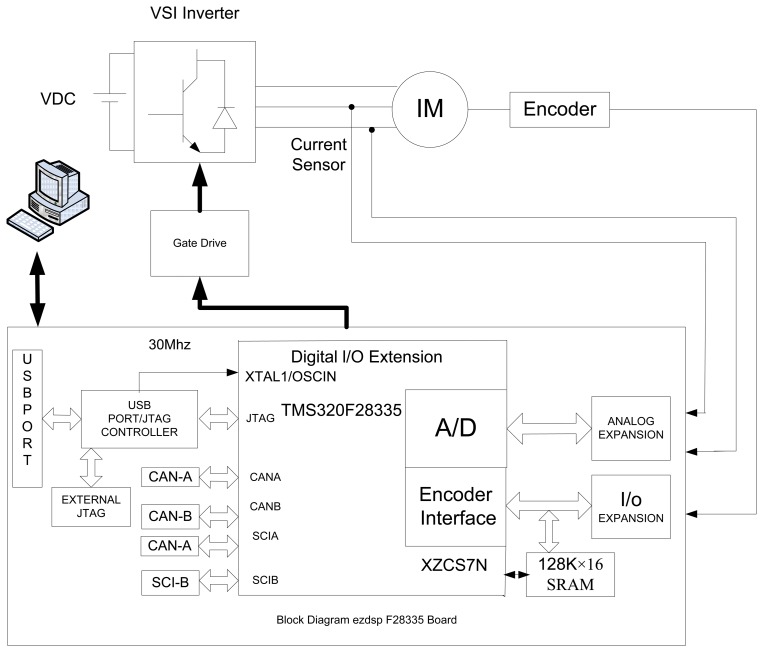
Block diagram of the hardware schematic for real-time implementation of VSI fed IM drive.

To implement the IM drive in real-time, an ezdspF28335 platform from Spectrum Digital (Stafford, TX, USA) is employed. Figure 14 shows a block diagram of the hardware schematic of a space vector modulated-voltage source inverter (SVM-VSI) fed IM drive. Three phase power inverters with 380 V DC bus voltage, 10 kHz SVM-PWM switching frequency and 2 μs dead time for short circuit protection are used to drive the IM.

As shown in Figure 14, the phase currents are sensed by the two HX 10-P/SP2 current transducer sensors, which have a current range of ±10 A. The current signals are fed back to the ezdspF28335 board through analogue to digital converter (A/D) channels. The output current signal of these sensors is converted to a voltage across the resistor connected (2 KΩ, 1/4 W) between the output terminal of the sensor and ground. These currents are fed to the DSP board through the A/D converter. Due to the noises from current sensors and A/D of the digital signal processor (DSP), there are lots of spikes in the motor currents, which cannot meet the requirements of vector control in the case of small signals. To remove these noises, there are some potential solutions such as using a suitable voltage source for the ADC conditioning circuit, a proper electronic design layout, using a small ceramic capacitor along with the electrolytic capacitor, using the proper op-amps [37–41], and using digital filters to omit the noise from motor current signals. In this work considering all of the aforementioned notices, a digital low pass filter with a suitable cut-off frequency (normally chosen above the chattering frequency) is employed. It is found that the best cut-off frequency is about 500 Hz.

**Figure 15.**
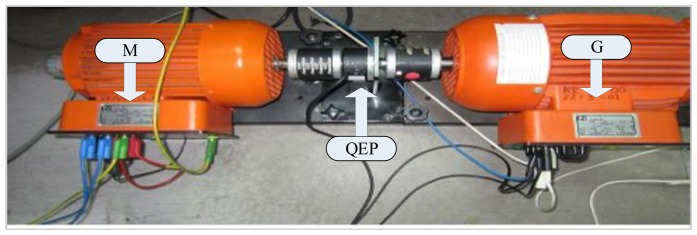
Connection of encoder for IM drive.

**Figure 16.**
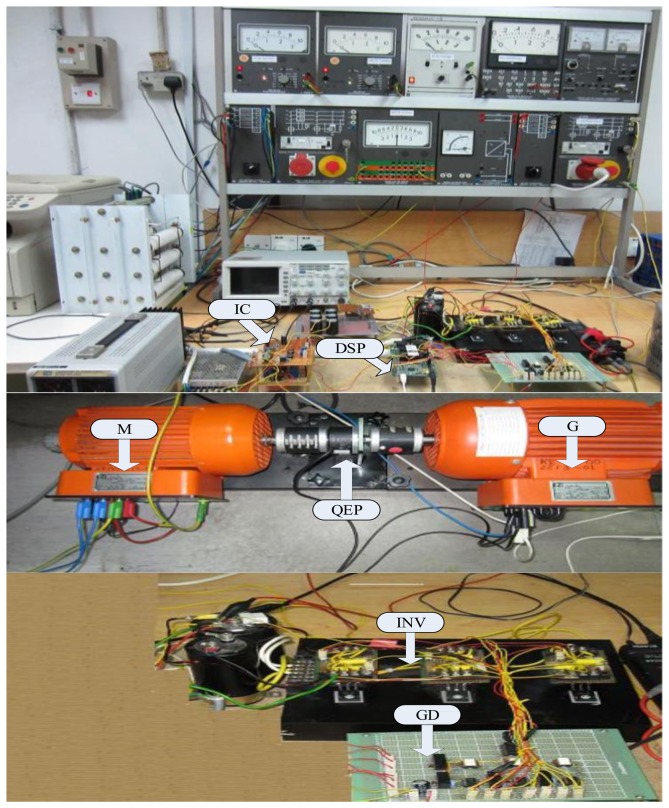
Experimental setup of the proposed IM drive.

Rotor position is sensed by an optical incremental encoder (QEP) E60H20 with three output channels (A, B, and index) which must be mounted properly on the rotor shaft as shown in Figure 15 and is fed back to the ezdspF28335 through the I/O expansion as seen in Figure 14. The used encoder generates 5000 pulses per turn which is multiplied by 4 using the quadrature mode, so the resolution of the encoder is 2*π*/(5,000 × 4) = 0.018°. A 16-bit position counter is used to count the encoder and it is reset in each revolution by an index pulse. Since the encoder is an open collector type with 15 V power supply, a 1 kΩ pull-up resistance is connected between the power supply and each output and some resistive voltage dividers are used to reduce the output voltages to 3.3 V to meet the DSP voltage conditions. This optical incremental encoder also used to calculate high-resolution speed measurements. It is noted that all related requirements for connection the sensors to DSP are provided with an interface circuit (IC) as shown in Figure 16.

The control algorithms are made by Simulink models and downloaded to the DSP board through Code Composer Studio (CCStudio) TI software. The outputs of the board are six logic signals, which are fed to the inverter (INV) through the gate drive (GD) circuit as shown in Figure 16. Due to the computational burden of the fuzzy controller, the sampling frequency of the proposed controller should be less than the PI controller in real-time to get the results properly. Thus, the sampling frequencies of experimental implementation are set at 10 kHz and 4 kHz for the tuned PI and the proposed controllers, respectively. The necessary data is saved on DSP's memory with 400 Hz sampling frequency. A DC generator is coupled with the IM as a load.

The experimental setup for the proposed NBLFC based prototype 1 kW IM drive system is shown in Figure 16.

### 5.3. Experimental Results and Discussion

The tuned PI, the conventional BLFC, and the proposed NBLFC were simulated to search for the controller which provides the best dynamic performance. Through simulation tests it was found that the proposed NBLFC provides the best dynamic performance. To validate the simulation results, the proposed controller is implemented in real-time and compared with the conventional BLFC and the tuned PI controller based IM drive. As the PI controller has been utilized widely in the industry, it was considered as the benchmark controller. For the experimental test verifications, the following cases are considered. If not mentioned, like in the simulation results, all other parameters are considered nominal in all the cases:

***Case-1***: Step changes in command speed with no load.
***Case-2***: Step increase in load from ‘0’ to full load.

**Figure 17.**
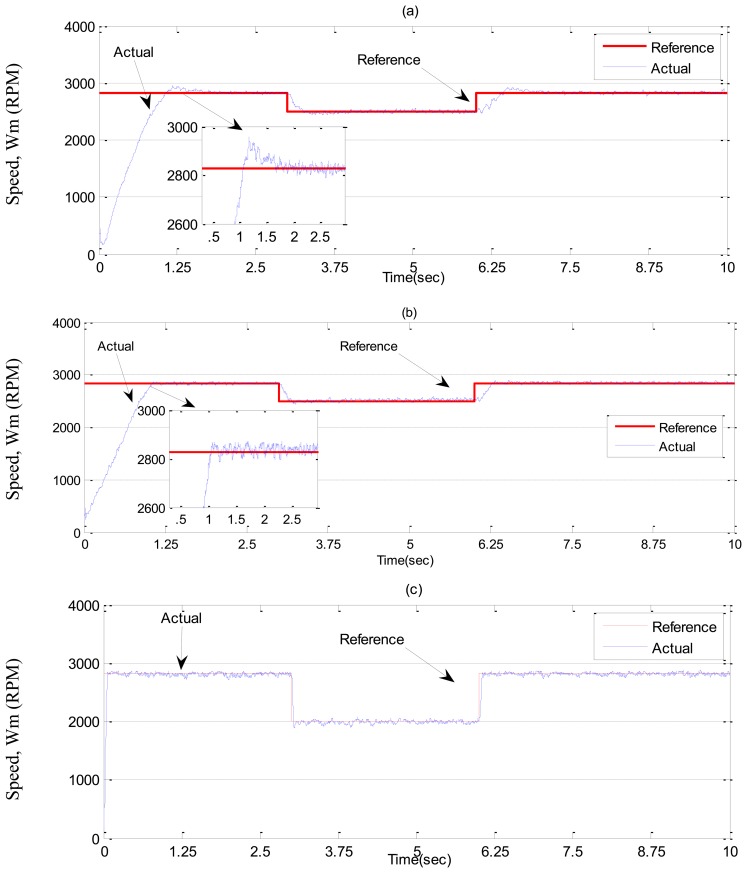
Experimental speed responses-based IM drive in *Case-1*; (**a**) the tuned PI controller, (**b**) the conventional BLFC controller, and (**c**) the proposed NBLFC controller.

**Figure 18.**
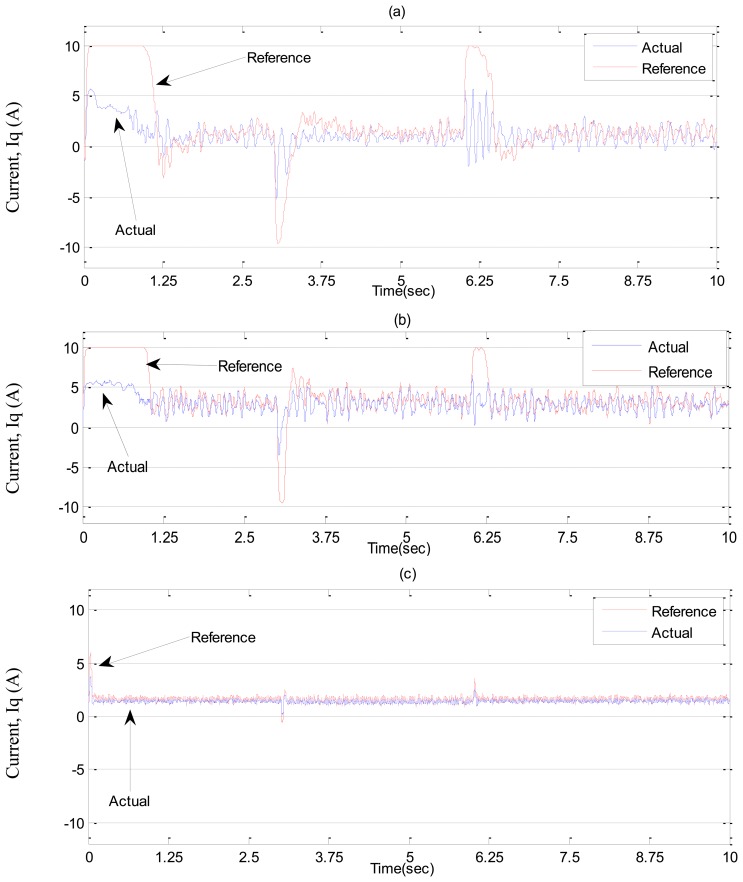
Experimental q-axis current responses based IM drive in *Case-1*; (**a**) the tuned PI controller, (**b**) the conventional BLFC controller, and (**c**) the proposed NBLFC controller.

It is noteworthy that the PI parameters in practice, are found as, Kp = 0.8, Ki = 0.0002, and Kc = 0.0002. In ***Case-1***, experimental results are illustrated in Figures 17 and 18 for the tuned PI and the proposed NBLFC based IM drive, respectively. From Figure 17, it can be seen that speed response of the conventional BLFC controller and the proposed NBLFC are better than that of the tuned PI controller in terms of rising, overshoot, and settling times with the step changes in command speeds. In addition, From Figure 17, it can be seen that dynamic performance of the proposed NBLFC controller is better than that of the conventional BLFC controller in terms of rising time. It can be seen from Figure 18 that the proposed NBLFC controller exhibit significantly less steady-state chattering in current as compared to the conventional BLFC and the tuned PI.

**Figure 19.**
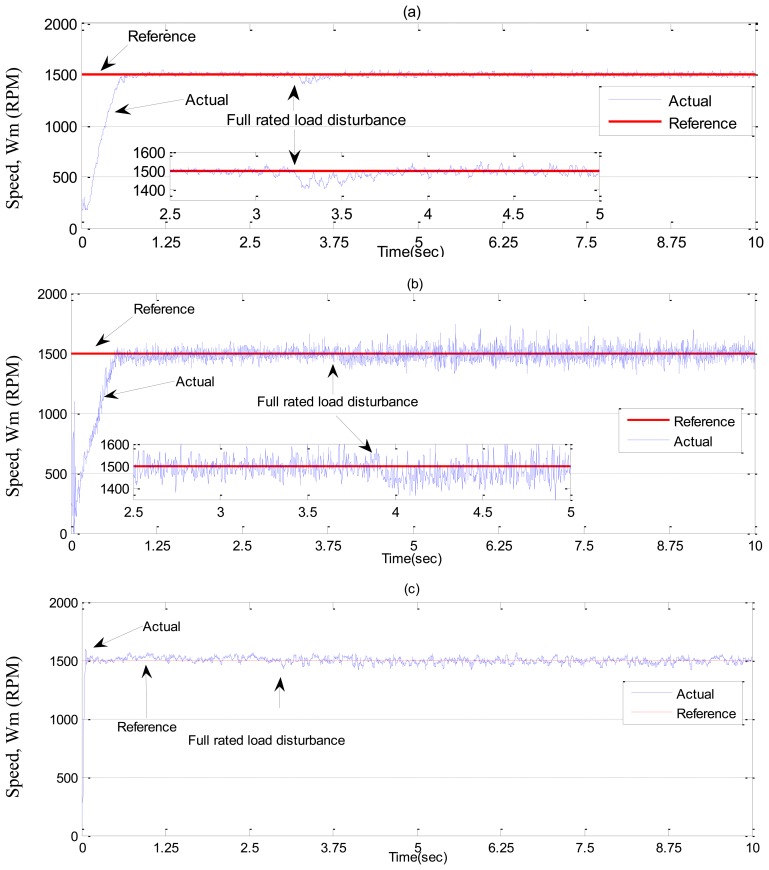
Experimental speed responses-based IM drive in *Case-2*; (**a**) the tuned PI controller, (**b**) the conventional BLFC controller, and (**c**) the proposed NBLFC controller.

**Figure 20.**
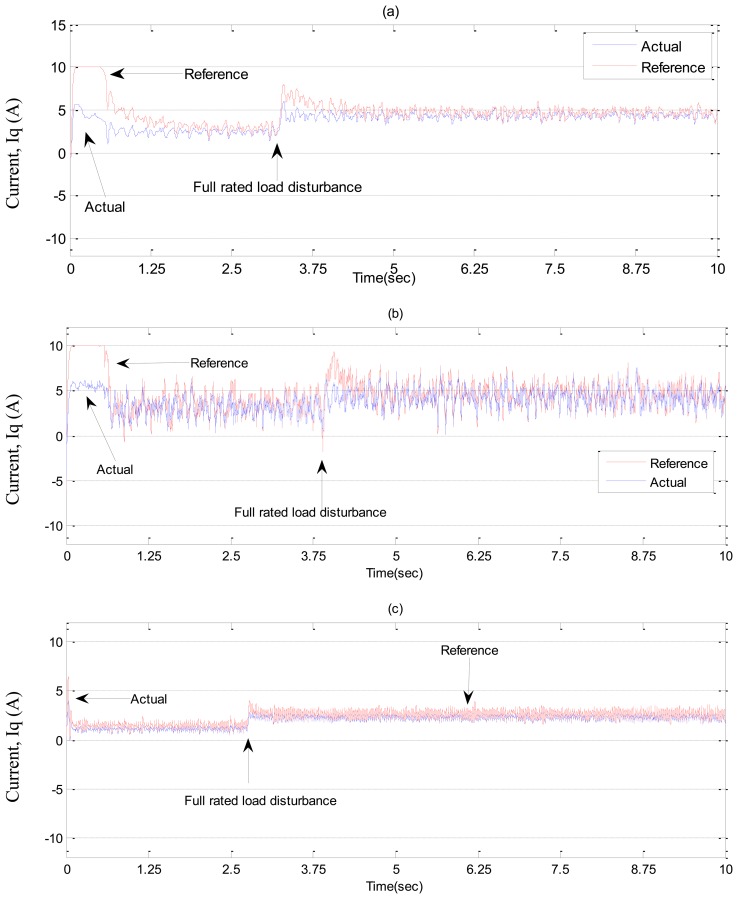
Experimental q-axis current responses-based IM drive in *Case-2*; (a) the tuned PI controller, (b) the conventional BLFC controller, and (c) the proposed NBLFC controller.

In ***Case-2***, experimental results are shown in Figures 19 and 20 for a step increase in full load. The motor was running under no load condition, then, suddenly a full load disturbance is applied to the motor. From these Figures, it can be seen the PI controller suffers from a significant dip in speed (≈100 RPM) when the step increase in load is applied. Moreover, the rising time for the proposed NBLFC based IM drive is faster than that of the conventional BLFC and the tuned PI controller. It is also found that the conventional BLFC suffers from chattering when the external load disturbance is applied, whereas, it is found from Figures 19 and 20 that the proposed NBLFC is not only insensitive to load variations, but also free from chattering in steady-state despite the large uncertainty in the system. Thus, the proposed NBLFC generates less harmonic loss and associated heat in the motor, which is a major issue in real-time.

## 6. Conclusions

A novel boundary layer fuzzy controller-based IFOC of an IM drive has been presented in this paper. The structure of the proposed controller is based on e smoothing out the control discontinuity in a thin boundary layer near the sliding surface. The proposed fuzzy system based on the variable boundary layer has been employed to adjust the thickness of boundary layer near the sliding surface to improve tracking performance under small uncertainties. To eliminate the chattering despite large uncertainties, an integral filter has been used in the variable thin boundary layer so that the stability of the proposed NBLFC is guaranteed. The proposed NBLFC-based IM drive has been successfully implemented in real-time using an ezdspF28335 DSP board and physical sensors for a prototype 1.5 HP motor. The appropriate utilization of the current and position sensors has been contemplated. The performance of the proposed NBLFC has been tested in both simulation and experiment. The performance of the proposed NBLFC controller was found superior to the conventional PI and SMC controllers under different operating conditions such as a step change in command speed, load disturbance and parameter variations over a wide speed range. Furthermore, the proposed NBLFC has reduced the steady-state chattering in current. Thus, the proposed NBLFC ensures less harmonic loss and associated heat dissipation in the motor.

## Figures and Tables

**Table 1.** Fuzzy rule based matrix for NBLFC.

	**Switching Variable Amplitude|S** (**t**)**|**						
Alteration of switching variable amplitude | Δs(t)|		Z	S	M	MB	L	VL
	Z	VL	VL	L	L	MB	MB
	S	VL	L	L	MB	MB	M
	M	L	L	MB	MB	M	M
	MB	L	MB	MB	M	M	S
	L	MB	MB	M	M	S	S
	VL	MB	L	M	S	S	Z

**Table 2.** Induction motor parameters.

**Parameters**	**Value**	**Parameters**	**Value**
Rated power	1,000 W	Rated torque	3.37 NM
*R_s_*	6	*J_r_*	0.0055 Kg.m^2
*R_r_*	5.72	*B*	0.001 Kg.m^2/s
*L_s_*	428.7e-3H	*p*	2
*L_r_*	428.7e-3H	Rated speed	2830 RPM
*L_m_*	416.6e-3H		

## Nomenclature

*B*: Friction factor
*C*: Sliding surface coefficient
DMC: Digital Motor control
DSP: Digital signal processor
*e*(*t*): Error between actual and command speed
G: Generator
GD: Gate drive
*h*: Positive constant for sliding surface
IC: Interface circuit
INV: Inverter
*i_a_*, *i_b_*, *i_c_*: Three phase current
*i_αs_*, *i_βs_*: Stationary frame stator currents
$i_{ds}^{e}$, $i_{qs}^{e}$: Synchronous frame stator currents
$i_{dr}^{e}$, $i_{qr}^{e}$: Synchronous frame rotor currents
*J_r_*: Inertia of rotor
*k_d_*(*t*): Desired control gain
*L*(*t*): Lumped uncertainty
*L_m_*: Mutual inductance
*L_s_*,*L_r_*: Stator and rotor inductance, respectively
M: Motor
*P*: Number of poles
QEP: Optical incremental encoder
RPM: Revolutions per minute
*R_s_*,*R_r_*: Stator and rotor resistance, respectively
Sat: Saturation function
*_S_*(*t*): Time-varying surface
*Sgn*: Sign function
SVM: Space vector modulation
*T_e_*: Electromagnetic torque
*T_L_*: Load torque
*t_reach_*: Reaching phase
*u*(*t*): Control effort
*u_eq_*(*t*): Equivalent control
*u_r_*(*t*): Reaching control
VSI: Voltage source inverter
*V*(*t*): Lyapunov function
*ν_αs_*, *ν_βs_*: Synchronous frame stator voltages
$v_{dr}^{e}$, $v_{qr}^{e}$: Synchronous frame rotor voltages
$v_{ds}^{e}$, $v_{qs}^{e}$: Synchronous frame stator voltages
*ẋ*: First derivative of *x*
*ẍ*: Second derivative of *x*
$\text{z}\overset{*}{x}$: Command value of *x*
|*x*|: Absolute value of *x*
$\varphi_{ds}^{e}$, $\varphi_{qs}^{e}$: Synchronous frame stator fluxes
$\varphi_{dr}^{e}$, $\varphi_{qr}^{e}$: Synchronous frame rotor fluxes
*p*: Differential operator
*ω_e_*, *ω*_r_: Synchronous speed and rotor speed, respectively
*ω*_sl_: Slip angular frequency
σ: Total leakage factor, $\sigma =1-L_{m}^{2}/L_{s}L_{r}$
*θ_r_*: Rotor position
*θ_e_*: Synchronous position
*ΔS*(*t*): Alteration of switching variable
*Δe*(*t*): Change of speed error
*τ*: Integral constant time of system
*ψ*(*t*): Boundary layer thickness
*η*: Positive constant of stability threshold
*υ*: Cut-off filter frequency
